# Higher Training Frequency Is Important for Gaining Muscular Strength Under Volume-Matched Training

**DOI:** 10.3389/fphys.2018.00744

**Published:** 2018-07-02

**Authors:** Eisuke Ochi, Masataka Maruo, Yosuke Tsuchiya, Naokata Ishii, Koji Miura, Kazushige Sasaki

**Affiliations:** ^1^Faculty of Bioscience and Applied Chemistry, Hosei University, Tokyo, Japan; ^2^Graduate School of Education, Okayama University, Okayama, Japan; ^3^Faculty of Modern Life, Teikyo Heisei University, Tokyo, Japan; ^4^Graduate School of Arts and Sciences, University of Tokyo, Tokyo, Japan; ^5^Department of Physical Education, International Pacific University, Okayama, Japan; ^6^Faculty of Human Sciences and Design, Japan Women’s University, Tokyo, Japan

**Keywords:** resistance training, muscle thickness, isometric strength, quadriceps, muscle stiffness

## Abstract

**Background:** This study investigated the effect of volume-matched strength training programs with different frequency and subsequent detraining on muscle size and strength.

**Methods:** During a training period of 11 weeks, untrained subjects (age: 22.3 ± 0.9 years, height: 173.1 ± 4.8 cm and body mass: 66.8 ± 8.4 kg) performed knee-extension exercise at 67% of their estimated one-repetition maximum either one session per week (T1 group: 6 sets of 12 repetitions per session; *n* = 10) or three sessions per week (T3 group: 2 sets of 12 repetitions per session; *n* = 10). Rating of perceived exertion (RPE) and muscle stiffness were measured as an index of muscle fatigue and muscle damage, respectively. The magnitude of muscle hypertrophy was assessed with thigh circumference and the quadriceps muscle thickness. The changes in muscle strength were measured with isometric maximum voluntary contraction torque (MVC).

**Results:** During the training period, RPE was significantly higher in the T1 than in the T3 (*p* < 0.001). After 11 weeks of training, both groups exhibited significant improvements in thigh circumference, muscle thickness, and MVC compared with baseline values. However, there was a significant group difference in MVC improvement at week 11 (T1: 43.5 ± 15.5%, T3: 65.2 ± 23.2%, *p* < 0.05). After 6 weeks of detraining, both groups showed the significant decreases in thigh circumference and muscle thickness from those at the end of training period, while no significant effect of detraining was observed in MVC.

**Conclusion:** These results suggest that three training sessions per week with two sets are recommended for untrained subjects to improve muscle strength while minimizing fatigue compared to one session per week with six sets.

## Introduction

Strength training being a safe and effective way to increase muscle size and strength, is supported by the American College of Sports Medicine (2009). While it is well established that the optimal strength training loading zone for gaining muscle size and strength corresponds to 8–12 repetitions maximum (RM) and 3–5 RM, respectively (American College of Sports Medicine, 2009), the effect of training frequency on subsequent neuromuscular adaptation is relatively under-researched.

In regards to training frequency, recent review papers have proposed that reducing the training volume and increasing the training frequency may be beneficial for muscle hypertrophy (Schoenfeld et al., 2016; Dankel et al., 2017). As earlier studies (McLester et al., 2000; Schoenfeld et al., 2015; Brigatto et al., 2018) have shown that training either once or three times a week in trained subjects elicited similar improvements in muscular strength and hypertrophy, these results suggest that the training frequency might not be of great importance in trained individuals. However, a meta-analysis by Schoenfeld et al. (2016) concluded that training twice a week resulted in superior muscle hypertrophy compared with training once a week in untrained subjects. As they also mentioned that “Due to an absence of data, it is not clear whether training muscle groups more than 3 days per week might enhance the hypertrophic response” (Schoenfeld et al., 2016), the importance of higher training frequency for muscular hypertrophy in untrained subjects is still unknown. A recent meta-analysis by Grgic et al. (2018) suggested that there is a dose-response relationship between training frequency and muscular strength gain. However, when the training volume was identical, they found no significant effect. Thus, it remains unclear whether training frequency has an effect on muscular strength gain under volume-matched conditions.

When strength training is stopped (i.e., detraining), muscle size and strength gradually decrease (Colliander and Tesch, 1992; Kubo et al., 2010). Some studies have investigated the effect of training intensity on the decrease in muscle size (Häkkinen et al., 1985; Jespersen et al., 2009; Kubo et al., 2010) and strength (Häkkinen and Komi, 1982; Häkkinen et al., 1985) during detraining, the results of which are inconsistent. As per our knowledge, no study has investigated the effect of strength training frequency on the maintenance of muscle size and strength during detraining, while controlling the total training volume. We assumed that a higher training frequency would maintain muscular strength under volume-matched training conditions because of greater motor learning.

In this study, therefore, we aimed to investigate the effects of two knee-extension training programs, which differed in training frequency but not in total training volume, on the changes in muscle size and strength during an 11-week training and a subsequent 6-week detraining period. We hypothesized that the two programs would result in different RPE during exercise, but similar adaptations to strength training and detraining.

## Materials and Methods

### Subjects

Twenty healthy male college students (age: 22.3 ± 0.9 years, height: 173.1 ± 4.8 cm, body mass: 66.8 ± 8.4 kg) participated in the study. The sample size was determined by a power analysis (G^∗^power, version 3.0.10, Heinrich-Heine University, Dusseldorf, Germany) by setting the effect size as 0.25, α level of 0.05 and power (1-β) of 0.80 for the comparison between groups, which showed that at least ten participants were necessary (Fink et al., 2017). They had not participated in any regular resistance training for at least 1 year prior to recruitment. Before participation, the subjects were given a detailed explanation of the study protocol, and each of them signed an informed consent form. The subjects were requested to avoid interventions such as massage, stretching, strenuous exercise, and excessive consumption of food and alcohol during the study period. Food and fluid intakes were not restricted. The study was conducted in accordance with the principles of the Declaration of Helsinki and was approved by the local ethics committee for human experiments at Juntendo University (ID: 24–39).

### Experimental Protocols

This study investigated the effect of two exercise training programs with different frequencies but same volume per week on muscle size and strength over an 11-week training followed by a 6-week detraining period. Twenty subjects were divided into a T1 group (*n* = 10), who trained once a week and a T3 group (*n* = 10), who trained three times a week. The subjects were randomly assigned to two groups using a table of random numbers in such a manner as to minimize the inter-group differences in age, body fat, body mass index. The total training volume per week was the same in both groups, with the number of sets in each training session being different (see section “Training Programs” for details). The following outcomes were measured before and after 3, 6, 9, and 11 weeks of training and after 3 and 6 weeks of detraining (denoted as weeks 14 and 17, respectively, in the ‘Figures’ and ‘Tables’): body composition, thigh circumference, muscle thickness, muscle stiffness, and maximum voluntary contraction torque (MVC). The measurements were made by a single investigator who was well-trained and blinded to the groups. During the training period, each measurement session was scheduled 72 h after the corresponding last training session, while the baseline measurement was performed 1 week before the first training session. Measurements were taken before each training session, with only RPE measured during each training session.

### Training Programs

Knee-extension exercise training with both legs was conducted using a leg extension machine (CYBEX, United States). The T1 group performed one training session (comprising 6 sets of 12 leg extensions at 67% of their estimated 1RM) per week, whereas the T3 group performed three sessions (each comprising 2 sets of 12 repetitions at 67% of estimated their 1RM) per week. This intensity was set according to the guideline of American College of Sports Medicine (2009). Both groups of subjects rested for 2 min between each set. The movement velocity was controlled using a metronome so that each 1 s knee-extension (concentric) and 1 s knee-flexion (eccentric) was followed by a 1 s rest period. The range of motion of the knee joint was from 90 to 0° (full extension). The machine’s backrest was set to an angle of 100° and the subject was secured with a belt at the waist to prevent sliding of the buttocks. If the subjects could not complete the prescribed repetitions because of fatigue, they were allowed a 2-min rest after which the remaining repetitions were performed to ensure a total of 72 repetitions (6 sets of 12 repetitions) per week.

To determine training intensity, each subject’s 1RM was estimated using the same machine as for training. If the subjects were able to smoothly perform five repetitions with a given weight load, the load was increased by 5 kg. Such trials were repeated with a 2-min rest between trials, until the subject could complete 4 but not 5 repetitions. We calculated estimated 1RM using the following formula: 1RM = 4 RM × 100/90. The 1RM was re-estimated before training sessions at 2, 4, 6, 8, and 10 weeks of training and after 2 and 5 weeks of detraining.

### Measurements

#### Rating of Perceived Exertion

Rating of perceived exertion was measured immediately after the knee-extension exercise in both groups using a psychophysical category scale (Noble et al., 1983) with the subject rating the strength of his perception from 0 (“no exertion at all”) to 10 (“extremely strong”) (Loenneke et al., 2011). The RPE test–retest reliability based on the coefficient of variation (CV) was 5.9%.

#### Muscle Stiffness

Using ultrasound shear-wave elastography, we measured the stiffness of the three heads of the left quadriceps muscles (Vastus Lateralis: VL, Rectus Femoris: RF, and Vastus Medialis: VM) with the subject standing upright. An ultrasonic scanner (Aixplorer version 4.2, Supersonic Imagine, France) was used in shear-wave elastography mode with musculoskeletal preset. An electronic linear-array probe (SL 15-4, Supersonic Imagine) coated with water-soluble transmission gel was placed longitudinally on each muscle head. Muscle shear modulus (μ), a measure of normalized muscle stiffness, was calculated using the following equation: μ = ρ*V*_s_^2^, where ρ is the density of muscle (assumed to be 1,000 kg/m^3^) and *V*_s_ is the velocity of shear-wave propagation caused by a focused ultrasound beam from the scanner. A 10 mm square map of the muscle shear modulus with a spatial resolution of 1 mm × 1 mm was obtained with each ultrasound image. A representative value of the shear modulus for each muscle head was then determined by the spatial averaging over a 5 mm diameter circle. The shear modulus of each muscle head was measured at 30, 50, and 70% of the distance between the greater trochanter and the lateral condyle, as marked for thigh circumference measurements, and the mean value of the three sites was calculated (Akagi et al., 2015). The test–retest reliability of the muscle stiffness measures based on the CV was 2.0%.

#### Thigh Circumference

The left thigh circumference was measured using a tape measure at 30, 50, and 70% of the distance from the greater trochanter point to the lateral condyle while the subject was standing upright. Measurement sites were marked with semi-permanent ink pen and maintained throughout the experimental period. The test–retest reliability of the circumference measures based on the CV was 2.3%.

#### Maximum Voluntary Contraction Torque

After a warm-up exercise consisting of 3–5 knee extensions, maximal voluntary isometric concentric contraction torque of the non-dominant (left) leg was measured by a custom-made knee-extension dynamometer, at a knee joint angle of 90° and hip joint angle of 70° flexion. Following sufficient familiarization and warm-up, subjects performed three 3 s MVC trials with a 60 s rest between trials. We calculated MVC as an average knee-extension torque over a 0.25 s epoch at the middle of contraction. The average MVC from the three trials was used for further analysis. The test–retest reliability of the MVC measures based on the CV was 3.8%.

#### Muscle Thickness

We measured the thickness of the four heads of the left quadriceps muscles (VL, RF, VM, and Vastus Intermedius: VI) with the same setup as used in the measurement of muscle stiffness (Akagi et al., 2015). Muscle thickness was measured at 30, 50, and 70% of the distance from the greater trochanter to the lateral condyle of the left thigh. We took precautions to keep the probe perpendicular to the skin and to apply minimal compression on the tissues during image acquisition. Measurement sites were marked at the first measurement, using a semi-permanent ink pen, which was then maintained throughout the experimental period by writing over each mark again on each training day. Three images for each site were obtained and the average recorded. We calculated the muscle thickness from each image as the distance from the bone to the subcutaneous fat. The test–retest reliability of the muscle thickness measures based on the CV was 1.7%.

### Statistical Analyses

All analyses were performed using SPSS Statistics software version 20.0 (IBM, Armonk, NY, United States). Data are expressed as mean ± standard deviation (SD). All parameters were expressed as absolute values, and MVC was expressed as both an absolute value and a value relative to the pre-training MVC (as a percentage). For each of the measurement items, two-way repeated measures analysis of variance (ANOVA) was used to test main effects of time and group as well as their interaction. When a significant main effect or interaction was found, Student’s *t*-test with Bonferroni correction was performed. A *p*-value of <0.05 was considered statistically significant. The magnitudes of the differences were calculated using standardized differences based on Cohen’s D units by means of effect sizes (ES) (Hopkins et al., 2009). Effect size (ES) was calculated as the pretest–post-test change, divided by the pooled pretest SD. The Cohen’s *D* results were qualitatively interpreted using the following thresholds: <0.2, trivial; 0.2–0.6, small; 0.6–1.2, moderate; 1.2–2.0, large; 2.0–4.0, very large; and >4.0, nearly perfect (Cohen, 1988).

## Results

### One-Repetition Maximum

Compared with the baseline (pre), both groups showed significant improvements in estimated 1RM after 2, 4, 6, 8, and 10 weeks of training and even after the subsequent 6-week detraining (T1: 12–53% T3: 15–53%, *p* < 0.01 for both groups, **Table 1**). No significant difference was observed between the groups at any time point (pre; ES = 0.55, 95% confidence interval (CI): -1.42 to 0.36, week 2; ES = 0.65, 95% CI: -0.55 to 0.25, week 4; ES = 0.72, 95% CI: -1.63 to 0.18, week 6; ES = 0.81, 95% CI: -1.68 to 0.14, week 8; ES = 0.54, 95% CI: -1.40 to 0.38, week 10; ES = 0.59, 95% CI: -1.45 to 0.33, week 14; ES = 0.53, 95% CI: -1.40 to 0.38, week 17; ES = 0.47, 95% CI: -1.33 to 0.44).

**Table 1:** Changes (mean ± SD) in 1 RM before (pre) and after strength training and detraining.

	Training						Detraining	
	Pre	Week 2	Week 4	Week 6	Week 8	Week 10	Week 14	Week 17
	Mean ± *SD*	Mean ± *SD*	Mean ± *SD*	Mean ± *SD*	Mean ± *SD*	Mean ± *SD*	Mean ± *SD*	Mean ± *SD*
**1 RM (kg)**								
T1 (*n* = 10)	96.7 ± 12.4	108.3 ± 16.2^∗∗^	117.4 ± 13.9^∗∗^	124.6 ± 11.5^∗∗^	136.0 ± 12.9^∗∗^	147.0 ± 18.0^∗∗^	144.7 ± 18.1^∗∗^	147.4 ± 20.7^∗∗^
T3 (*n* = 10)	106.3 ± 21.3	121.7 ± 22.6^∗∗^	134.6 ± 28.9^∗∗^	141.5 ± 27.2^∗∗^	147.5 ± 27.4^∗∗^	160.8 ± 28.0^∗∗^	156.9 ± 27.0^∗∗^	161.0 ± 35.6^∗∗^

*^∗∗^Within-group significant differences from before training, p < 0.01.*

### Rating of Perceived Exertion

**FIGURE 1:**
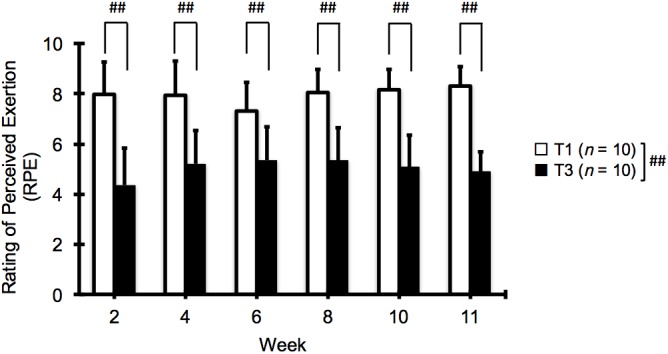
Changes in ratings of perceived exertion (RPE) at 2, 4, 6, 8, 10, and 11 weeks of low (T1) and high (T3) frequency training. Data are expressed as mean ± SD (*n* = 10 in each group). ^##^*p* < 0.01 for the difference between T1 and T3.

The T1 group reported a higher RPE than the T3 group at all time points (*p* < 0.01, **Figure 1**, week 2; ES = 2.62, 95% CI: 1.33 to 3.67, week 4; ES = 2.04, 95% CI: 0.89 to 3.02, week 6; ES = 1.56, 95% CI: 0.50 to 2.48, week 8; ES = 2.44, 95% CI: 1.20 to 3.47, week 10; ES = 2.89, 95% CI: 1.54 to 3.99, week 11; ES = 4.27, 95% CI: 2.54 to 5.62). No significant change in RPE was observed in either group throughout the training period.

### Muscle Stiffness

There was no significant change in muscle stiffness (shear modulus) in either group and thus no significant difference between the groups at any time point (**Table 2**).

**Table 2:** Changes (mean ± SD) in muscle stiffness (shear modulus) before (pre) and after strength training and detraining.

	Training					Detraining	
	Pre	Week 3	Week 6	Week 9	Week 11	Week 14	Week 17
	Mean ± *SD*	Mean ± *SD*	Mean ± *SD*	Mean ± *SD*	Mean ± *SD*	Mean ± *SD*	Mean ± *SD*
**VL (kPa)**							
T1 (*n* = 10)	10.7 ± 3.7	9.2 ± 2.6	11.9 ± 2.9	10.7 ± 2.0	8.4 ± 3.5	8.9 ± 3.1	7.5 ± 4.2
T3 (*n* = 10)	11.9 ± 5.2	10.2 ± 3.9	13.0 ± 4.6	10.3 ± 3.8	9.6 ± 2.9	9.8 ± 3.8	8.7 ± 2.5
**RF (kPa)**							
T1 (*n* = 10)	8.5 ± 1.7	9.0 ± 2.7	10.1 ± 2.8	9.2 ± 3.0	9.1 ± 1.8	8.5 ± 2.4	7.3 ± 2.3
T3 (*n* = 10)	9.2 ± 2.9	8.8 ± 1.7	11.0 ± 2.4	9.7 ± 2.9	7.8 ± 1.9	9.1 ± 2.9	9.3 ± 3.0
**VM (kPa)**							
T1 (*n* = 10)	8.2 ± 3.6	7.1 ± 1.3	9.8 ± 3.1	9.6 ± 1.8	7.7 ± 2.3	7.5 ± 2.5	6.7 ± 1.8
T3 (*n* = 10)	9.9 ± 2.5	8.1 ± 2.3	10.4 ± 3.0	9.1 ± 2.9	9.0 ± 3.7	8.3 ± 2.7	8.4 ± 2.3

*VL, Vastus Lateralis; RF, Rectus Femoris; VM, Vastus Medialis.*

### Thigh Circumference

Compared with the baseline (pre), both groups showed significant increases in thigh circumference after 9 and 11 weeks of training measured at 30 and 50% (T1: 2.6–6.7% T3: 2.8–3.8%, *p* < 0.01 for both groups, **Table 3**) but not at 70% of the thigh length. No significant difference was found between the groups at any time point.

**Table 3:** Changes (mean ± SD) in thigh circumstance, and MVC before (pre) and after strength training and detraining.

	Training					Detraining	
	Pre	Week 3	Week 6	Week 9	Week 11	Week 14	Week 17
	Mean ± *SD*	Mean ± *SD*	Mean ± *SD*	Mean ± *SD*	Mean ± *SD*	Mean ± *SD*	Mean ± *SD*
**MVC (Nm)**							
T1 (*n* = 10)	153.0 ± 29.7	174.4 ± 43.7^∗∗^	190.4 ± 49.9^∗∗^	201.5 ± 52.6^∗∗^	221.3 ± 56.7^∗∗^	214.9 ± 57.1^∗∗^	214.8 ± 57.6^∗∗^
T3 (*n* = 10)	169.4 ± 47.3	196.6 ± 55.0^∗∗^	219.4 ± 56.0^∗∗^	238.0 ± 53.7^∗∗^	270.7 ± 47.7^∗∗^^†^	271.0 ± 56.3^∗∗^	254.9 ± 53.7^∗∗^
**Circumference**							
**30% (cm)**							
T1 (*n* = 10)	52.0 ± 2.4	52.6 ± 2.8	52.6 ± 2.7	53.7 ± 2.7^∗∗^	53.7 ± 2.4^∗∗^	53.5 ± 2.5	52.8 ± 2.8
T3 (*n* = 10)	55.1 ± 4.3	55.8 ± 4.3	56.2 ± 4.0	56.6 ± 4.0^∗∗^	56.7 ± 4.1^∗∗^	56.5 ± 4.4	55.9 ± 4.4
**50% (cm)**							
T1 (*n* = 10)	49.5 ± 1.9	49.8 ± 2.0	50.4 ± 1.6	50.8 ± 1.6^∗∗^	50.9 ± 1.6^∗∗^	50.8 ± 1.8	50.0 ± 2.4
T3 (*n* = 10)	51.6 ± 4.3	52.3 ± 4.1	52.5 ± 3.8	52.9 ± 4.0^∗∗^	53.3 ± 4.1^∗∗^	53.4 ± 4.3	53.1 ± 4.5
**70% (cm)**							
T1 (*n* = 10)	43.0 ± 1.9	43.3 ± 2.2	45.2 ± 1.9	45.6 ± 1.5	45.8 ± 1.6	45.5 ± 1.9	44.5 ± 2.2
T3 (*n* = 10)	45.6 ± 4.2	46.1 ± 4.1	46.6 ± 3.7	47.1 ± 4.0	47.6 ± 4.1	47.3 ± 4.3	47.0 ± 4.3

*^∗∗^ Within-group significant differences from before training, p < 0.01.*

*^†^ Significant differences from T1, p < 0.05.*

### Maximum Voluntary Contraction Torque

**FIGURE 2:**
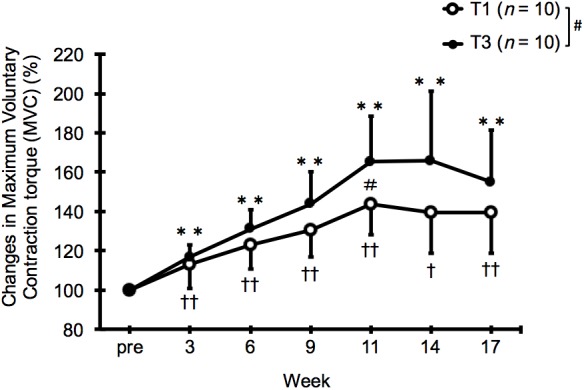
Relative changes in maximum voluntary contraction torque (MVC) from baseline (pre) after 11 weeks of low (T1) and high (T3) frequency training and subsequent 6 weeks of detraining. Data are expressed as mean ± SD (*n* = 10 in each group). ^∗∗^*p* < 0.01 compared with the pre-training value for T3. ^†^*p* < 0.05, ^††^*p* < 0.01 compared with the pre-training value for T1. ^#^*p* < 0.05 for the difference between T1 and T3.

Compared with the baseline (pre), both groups showed significant improvements in MVC after 3, 6, 9, and 11 weeks of training (*p* < 0.01) and even after the subsequent 6-week detraining (*p* < 0.01–0.05 at weeks 14 and 17, **Figure 2** and **Table 3**). A significant interaction effect was observed, and the improvement in MVC was significantly greater in the T3 group than in the T1 group at week 11 (T1: 43.5 ± 15.5%, T3: 65.2 ± 23.2%, *p* < 0.05, pre; ES = -0.02, 95% CI: -1.28 to 0.49, week 3; ES = 0.45, 95% CI: -1.31 to 0.46, week 6; ES = 0.55, 95% CI: -1.41 to 0.37, week 9; ES = 0.69, 95% CI: -1.55 to 0.25, week 11; ES = 0.94, 95% CI: -1.82 to -0.02, week 14; ES = 0.99, 95% CI: -1.87 to 0.02, week 17; ES = 0.72, 95% CI: -1.59 to 0.22).

### Muscle Thickness

Compared with the baseline (pre), both groups showed significant increase in thickness of all the quadriceps muscles after 6, 9, and 11 weeks of training and even after the subsequent 3-week detraining (*p* < 0.01–0.05 at week 14, **Table 4**). Compared with the end of training (at week 11), however, muscle thickness significantly decreased during detraining in both groups (*p* < 0.01–0.05 at week 17, **Table 4**). No significant difference was observed between the groups at any time point.

**Table 4:** Changes (mean ± SD) in muscle thickness before (pre) and after strength training and detraining.

	Training					Detraining	
	Pre	Week 3	Week 6	Week 9	Week 11	Week 14	Week 17
	Mean ± *SD*	Mean ± *SD*	Mean ± *SD*	Mean ± *SD*	Mean ± *SD*	Mean ± *SD*	Mean ± *SD*
**VL (cm)**							
T1 (*n* = 10)	2.27 ± 0.24	2.36 ± 0.21	2.45 ± 0.19^∗∗^	2.49 ± 0.20^∗∗^	2.57 ± 0.20^∗∗^	2.46 ± 0.21^∗∗##^	2.38 ± 0.23^##^
T3 (*n* = 10)	2.28 ± 0.38	2.36 ± 0.37	2.43 ± 0.35^∗∗^	2.49 ± 0.35^∗∗^	2.58 ± 0.39^∗∗^	2.49 ± 0.39^∗∗##^	2.40 ± 0.38^##^
**RF (cm)**							
T1 (*n* = 10)	2.39 ± 0.24	2.50 ± 0.26	2.61 ± 0.28^∗∗^	2.66 ± 0.26^∗∗^	2.76 ± 0.26^∗∗^	2.67 ± 0.25^∗∗##^	2.54 ± 0.26^##^
T3 (*n* = 10)	2.32 ± 0.25	2.44 ± 0.26	2.54 ± 0.26^∗∗^	2.62 ± 0.26^∗∗^	2.71 ± 0.26^∗∗^	2.57 ± 0.24^∗∗##^	2.50 ± 0.22^∗∗##^
**VM (cm)**							
T1 (*n* = 10)	4.59 ± 0.49	4.78 ± 0.46	4.92 ± 0.45^∗∗^	4.98 ± 0.43^∗∗^	5.08 ± 0.44^∗∗^	5.04 ± 0.46^∗∗^	4.96 ± 0.44^∗#^
T3 (*n* = 10)	4.83 ± 0.71	5.04 ± 0.73	5.12 ± 0.73^∗∗^	5.22 ± 0.74^∗∗^	5.30 ± 0.76^∗∗^	5.20 ± 0.75^∗∗#^	5.13 ± 0.72^##^
**VI (cm)**							
T1 (*n* = 10)	2.53 ± 0.33	2.65 ± 0.28	2.73 ± 0.32^∗^	2.76 ± 0.33^∗∗^	2.82 ± 0.36^∗∗^	2.75 ± 0.37^∗∗#^	2.68 ± 0.39^##^
T3 (*n* = 10)	2.72 ± 0.61	2.90 ± 0.65	2.94 ± 0.67^∗∗^	2.99 ± 0.66^∗∗^	3.04 ± 0.70^∗∗^	2.99 ± 0.67^∗∗^	2.92 ± 0.65^##^

*^∗^Within-group significant differences from before training, p < 0.05. ^∗∗^p < 0.01.*

*^#^Within-group significant differences from 11 weeks training, p < 0.05. ^##^p < 0.01.*

*VL, Vastus Lateralis; RF, Rectus Femoris; VM, Vastus Medialis; VI, Vastus Intermedius.*

## Discussion

In this study, we investigated the effects of two volume-matched knee-extension strength training programs with different frequencies (once vs. three times a week), on the changes in muscle size and strength. After 11 weeks of training, both groups showed significant increases in thigh circumference, muscle thickness, estimated 1RM, and MVC compared with baseline (pre), while RPE during exercise was significantly higher in the T1 group than in the T3 group. Moreover, the improvement in MVC was significantly greater in the T3 group than in the T1 group at week 11 (i.e., the end of training period). After 6 weeks of detraining, muscle thickness significantly decreased from the value at week 11 in both groups, with no significant group difference.

Both the T1 and T3 groups showed significant increase in thickness of all the quadriceps muscles after 6 weeks of training. Recently, Damas et al. (2016) have provided evidence that an increase in muscle cross-sectional area could be due to swelling. Since we had to set a relatively short interval (3 days) from the last training session to the measurement because of the training schedule, the increase in muscle thickness observed in this study could overestimate the actual magnitude of muscle hypertrophy. However, we believe that such an overestimation does not invalidate our conclusion, because the muscle stiffness measured with ultrasound elastography, an index of exercise-induced muscle damage (Lacourpaille et al., 2014), did not change significantly in either group throughout the experimental period. In fact, we also confirmed that the value of muscle stiffness 3 days after the last training session was consistently similar between the groups despite the differences in the number of sets in each session (**Table 3**).

There was no significant difference in muscle thickness between groups. This is consistent with the results of previous studies (Candow and Burke, 2007; Arazi and Asadi, 2011; Gentil et al., 2015), which showed no significant differences in muscle mass for untrained subjects. Since Schoenfeld et al. (2016) suggested the importance of conducting research involving training muscle groups more than 3 days per week, our finding that the magnitude of muscle hypertrophy is primarily determined by total training volume (not by frequency) is valuable. However, RPE, the perceptual response to exercise, was significantly higher in the T1 group than that in the T3 group at all time points (**Figure 1**). The mean RPE values were 7.3–8.3 and 4.3–5.3 in the T1 and T3 groups, respectively. Using the same 0–10 category scale as in the present study, a previous study showed that RPEs immediately after three sets of eight repetitions of knee-extension exercise were 5.1–6.2 (Martín-Hernández et al., 2017). Therefore, we can reasonably assume that the difference in RPE between the T3 (two sets) and T1 (six sets) groups reflects the difference in the number of sets per training session and the magnitude of muscle fatigue.

In both groups, MVC increased to a greater extent (T1: 40% and T3: 55%) than did muscle thickness (T1: 10.9–15.6% and T3: 9.8–16.9%) after 11 weeks of strength training. This observation is in good agreement with those of the previous studies (Moritani and deVries, 1979; Narici et al., 1996; Ferri et al., 2003). Interestingly, we found that the change in MVC was significantly greater in the T3 group than in the T1 group at the end of the training period. This difference cannot be explained by the difference in training-induced muscle hypertrophy, because of the similar change in muscle thickness as mentioned above. We speculate that the T1 group may have had a greater expression of extra-cellular matrix proteins (such as type I collagen) in the trained muscle, which may have resulted in a decrease in the muscle specific tension (strength per unit cross-sectional area). Interestingly, the muscle strength of the human quadriceps muscles has been shown to negatively correlate with ultrasound echo intensity, an index of the amount of non-contractile tissue within the muscle (Rech et al., 2014; Wilhelm et al., 2014). In addition, it can be speculated that the superior strength results for the T3 group could be due to a greater motor learning effect following the extra weekly “practice.” However, this would be contrary to the recent meta-analysis by Grgic et al. (2018), wherein it was found that frequency was not a factor in strength development when training programs were matched for volume. We believe that the experimental conditions in the present study, including the single joint exercise and non-failure among young untrained males, could explain the results. Further studies are warranted to verify these suggestions.

As far as we know, this is the first study to investigate the effect of volume-matched strength training frequency on the changes in muscle size and strength during subsequent detraining. The results showed that during detraining, the muscle thickness decreased from the value at the end of training period in both groups. On the other hand, the isometric strength did not change significantly during the 6-week detraining period in either group. These results are generally consistent with those of the previous studies showing that the rate of strength loss during detraining was much lower than that of strength gain during the preceding training period (Colliander and Tesch, 1992; Staron et al., 1994; Ivey et al., 2000). Although not statistically significant, the group difference in MVC improvement observed at the end of the training period tended to be maintained during the detraining period (**Table 3**). As the MVC in this study was isometric, we suggest that isokinetic (or isotonic) MVC testing may clarify the results. This finding suggests that for a given total training volume, increasing frequency would be more advantageous for developing and maintaining muscle strength in untrained subjects than increasing the volume (number of sets) per session.

This study has several limitations. First, we did not have a control group and did not determine the nutritional status. Thus the true effectiveness of the training programs cannot be evaluated properly. This is particularly relevant to the changes in MVC following strength training and detraining, as a motor learning effect associated with repeated measurements could contribute in part to the strength improvement. Second, as our findings are specific to young untrained men, they cannot be applied to other populations such as trained individuals, female individuals, or elderly subjects. Third, although we observed an increase in muscle strength and thickness in both groups, the training intensity (67% 1RM) was relatively low in this study. Therefore, we believe that the subjects did not train to failure. Since we think that the subjects did not reach to failure, we suggest that they could have resulted in different adaptations when they have trained to failure. Forth, because 1RM was estimated from 4RM, it is difficult to understand actual 1RM and compare the changes with those of the other parameters such as MVC. Finally, the detraining period (6 weeks) was short compared to that in previous studies (Colliander and Tesch, 1992; Staron et al., 1994; Ivey et al., 2000), and we failed to observe a significant decrease in muscle strength. Further studies need to be conducted to investigate these limitations.

In summary, this study demonstrated that 2 sets of 12 repetitions (at 67% 1RM), performed three times a week were less strenuous and equally or more effective for gaining muscle size and strength in untrained subjects than were 6 sets of 12 repetitions performed once a week. Although we acknowledge that our findings have the potential to change with the use of a different percentage of 1RM and the number of repetitions and sets, we suggest that high-frequency training is preferable to low-frequency training in untrained subjects, as long as the total training volume is constant. We propose that training frequency should be prioritized when designing strength-training programs for untrained subjects, such that a target amount of exercise (load × repetitions × sets) per week is divided into three sessions.

## Author Contributions

EO, MM, YT, NI, KM, and KS conceived the study. EO, MM, and NI participated in the design and coordination of the study. YT and KM carried out the data collection and performed the statistical analysis. EO, MM, and KS helped to draft the manuscript. All authors read and approved the final manuscript.

## Conflict of Interest Statement

The authors declare that the research was conducted in the absence of any commercial or financial relationships that could be construed as a potential conflict of interest.
